# Supplementary material for: The Ies6 subunit is essential for INO80-mediated nucleosome organization
Source: Sci Rep. 2026 Feb 21;16:7466. doi: 10.1038/s41598-026-40504-8 (PMC12929563; doi:10.1038/s41598-026-40504-8)
Supplement: Supplementary file 1 — Supplementary Material 1 [file 41598_2026_40504_MOESM1_ESM.pdf]

SUPPLEMENTARY INFORMATION

The Ies6 subunit is essential for INO80-mediated nucleosome organization

Ashish Kumar Singh[1,2,3], Felix Mueller-Planitz[1]

1. Institute of Physiological Chemistry, Faculty of Medicine Carl Gustav Carus, Technische Universität Dresden, Fetscherstraße 74, 01307 Dresden, Germany

2. Molecular Biology, Biomedical Center, Faculty of Medicine, Ludwig-Maximilians-Universität München, 82152 Planegg-Martinsried, Germany

3. Present address: Epigenomics, Proliferation, and the Identity of Cells, Department of Developmental and Stem Cell Biology, Institut Pasteur, CNRS UMR3738, 75015 Paris, France

Supp. Table 1

Strain	Genotype
W1588-4C (yFMP013)	*MATa ade2-1 his3-11,15 leu2-3,112 trp1-1 ura3-1 can1-100 RAD5+*
YTT227 (yFMP014)	*MATa ade2-1 his3-11,15 leu2-3,112 trp1-1 ura3-1 can1-100 RAD5+ isw1Δ:ADE2 isw2Δ::LEU2 chd1Δ::TRP1*
yFMP627	*MATa ade2-1 his3-11,15 leu2-3,112 trp1-1 ura3-1 can1-100 RAD5+ ies6Δ::NATMX6*
yFMP628	*MATalpha ade2-1 his3-11,15 leu2-3,112 trp1-1 ura3-1 can1-100 RAD5+ ies6Δ::NATMX6*
yFMP468	*MATa/MATalpha ade2-1/ade2-1 his3-11,15/his3-11,15 leu2-3,112/leu2-3,112 trp1-1/trp1-1 ura3-1/ura3-1 can1-100/can1-100 RAD5+/RAD5+ IES6/ies6Δ::NATMX6 C1*
yFMP469	*MATa/MATalpha ade2-1/ade2-1 his3-11,15/his3-11,15 leu2-3,112/leu2-3,112 trp1-1/trp1-1 ura3-1/ura3-1 can1-100/can1-100 RAD5+/RAD5+ IES6/ies6Δ::NATMX6 C2*

List of yeast strains

Supp. Fig. S1

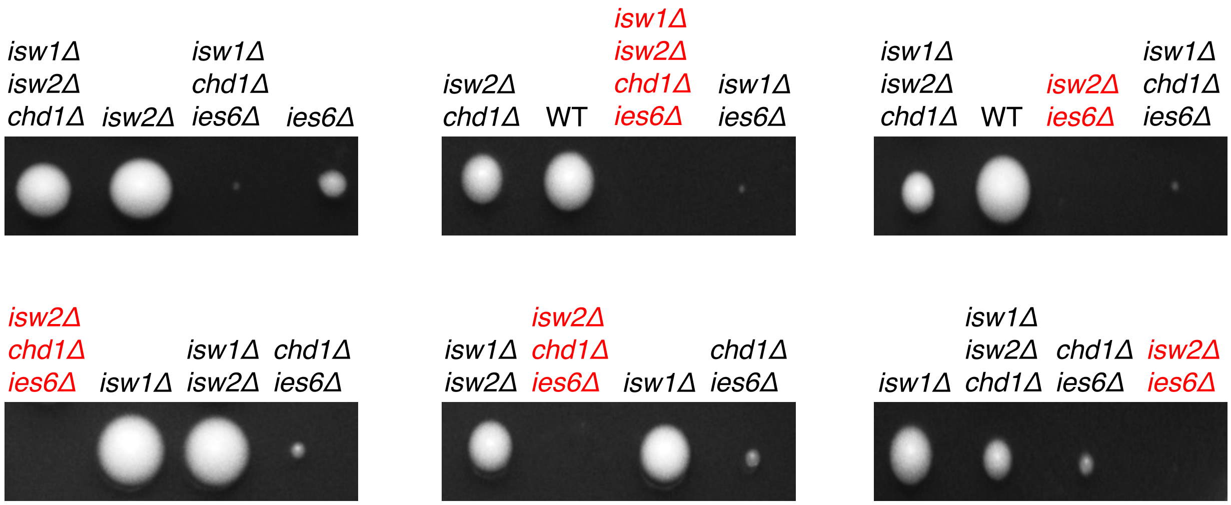

Further examples of tetrad dissection of diploids arising from TKO x *ies6Δ*. Tetrads were dissected on a YPAD plate, colonies grown for five days and replica-plated on minimal media lacking appropriate amino acids or on full media with antibiotic selection to identify genes lacking in the colony.

Supp. Fig. S2

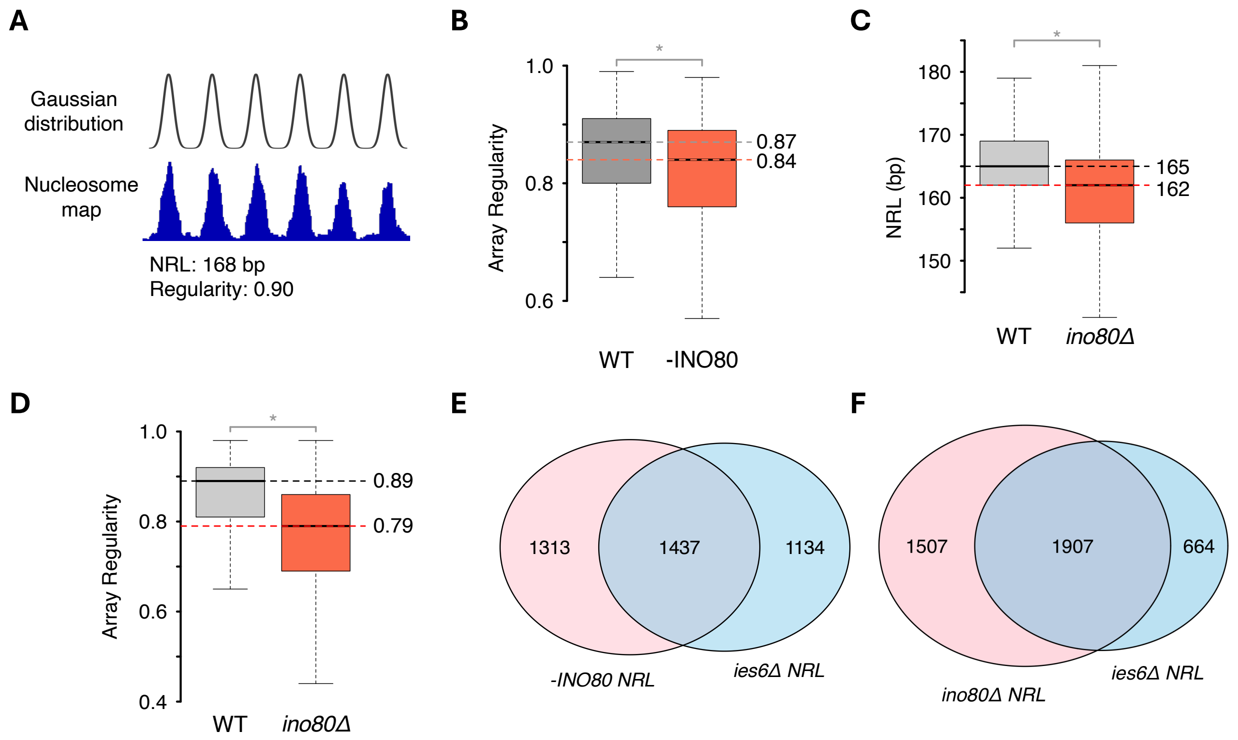

Deletion of Ino80 ATPase leads to lower NRL and array regularity in *S. cerevisiae*. (**A**) Fitting MNase-seq data over the TOF1 gene with a Gaussian pattern of varying repeat lengths to estimate NRL and array regularity at the gene. The NRL is obtained through the best-fitting Gaussian pattern, and the correlation score of the fit is used as a proxy of array regularity. (**B**) Boxplots showing array regularity distribution in 5015 yeast genes in WT and W303 cells depleted of INO80 via anchor-away method [1]. (**C**) Boxplots showing NRL distribution in 5015 yeast genes in WT and BY4741 cells lacking Ino80 ATPase. (**D**) Same as B, but for array regularity. Horizontal dotted line indicates the median NRL or array regularity in WT (gray) and cells lacking INO80 (red shades) cells. (**E**) Venn diagram showing overlap of genes with altered NRL in *ies6Δ* and cells depleted of INO80 in W303 background [1]. (**F**) Venn diagram showing overlap of genes with altered NRL in *ies6Δ* and *ino80Δ* cells [2].

Supp. Fig. S3

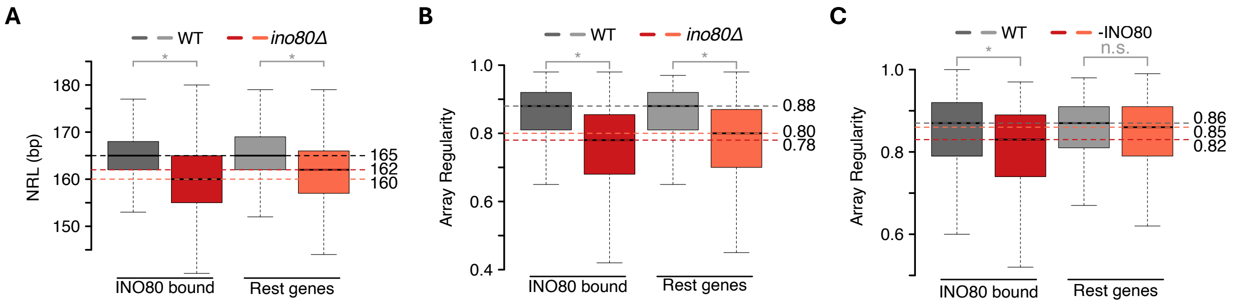

INO80-bound genes exhibit a greater reduction in NRL and array regularity than other genes following ablation of Ino80. (**A**) Boxplots showing NRL distribution in 1646 INO80 bound (dark shades) and rest (light shades) in WT and *ino80Δ* cells [2]. (**B**) Same as (A), but for array regularity. (**C**) Same as (A), but for array regularity distribution obtained after INO80 depletion [1]. Horizontal dotted line indicates the median NRL or array regularity in WT (gray) and INO80 lacking cells (red shades).

Supp. Fig. S4

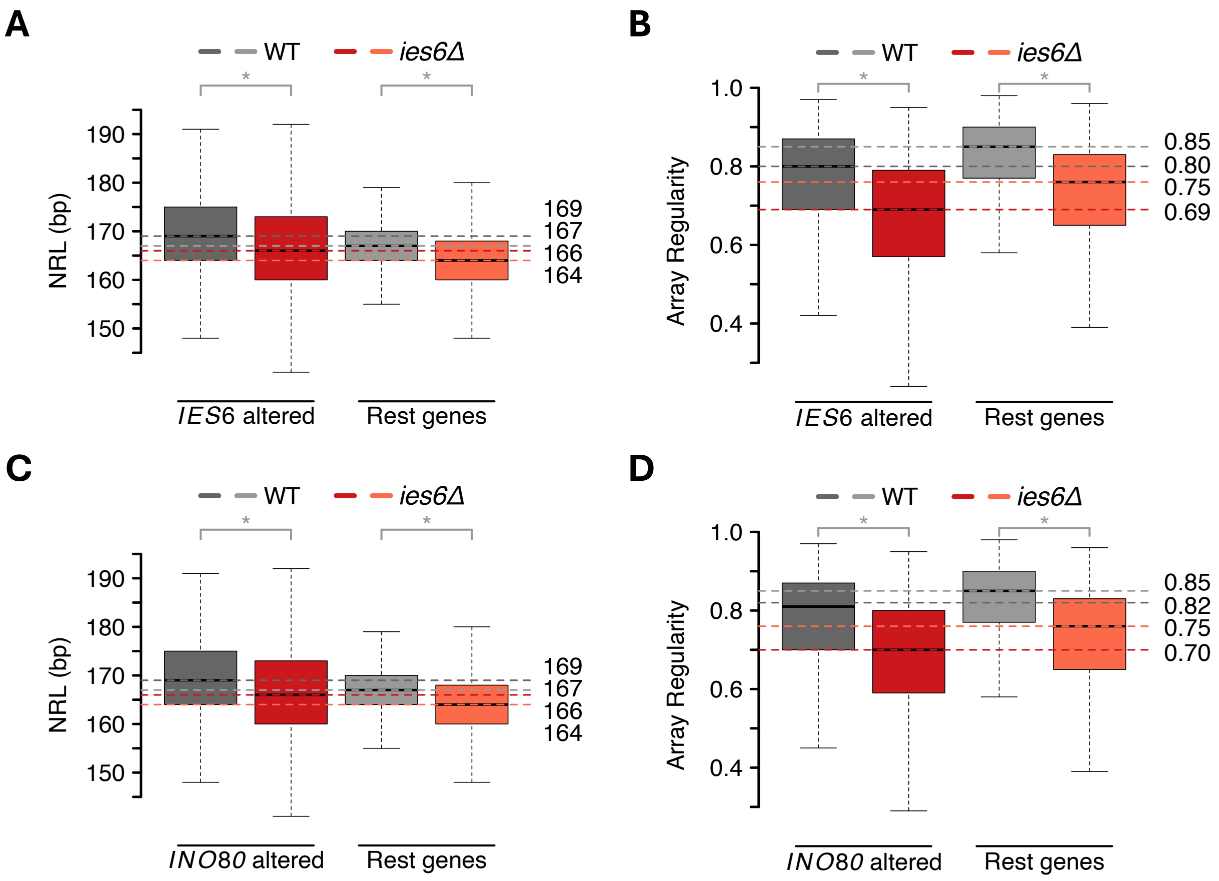

Genes with altered expression do not show different INO80 activity compared to unaltered genes in *ies6Δ* cells. (**A**) Boxplots showing NRL distribution in *ies6Δ* cells for all 1239 genes with at least 1.5-fold change in gene expression in *ies6Δ* cells (left) and remaining genes (right) in the indicated yeast strains. List of differentially expressed genes was obtained from [3]. (**B**) Boxplots showing array regularity distribution in the same set of genes as in (A). (**C**) Boxplots showing NRL distribution in *ies6Δ* cells for all 1349 genes with at least 1.5-fold change in gene expression in *ino80Δ* cells (left) and rest (right) of the genes in the indicated yeast strains. (**D**) Boxplots showing array regularity distribution in the same set of genes as in (C). Horizontal dotted line indicates the median NRL or array regularity in WT (gray shades) and *ies6Δ* (red shades) cells.

Supp. Fig. S5

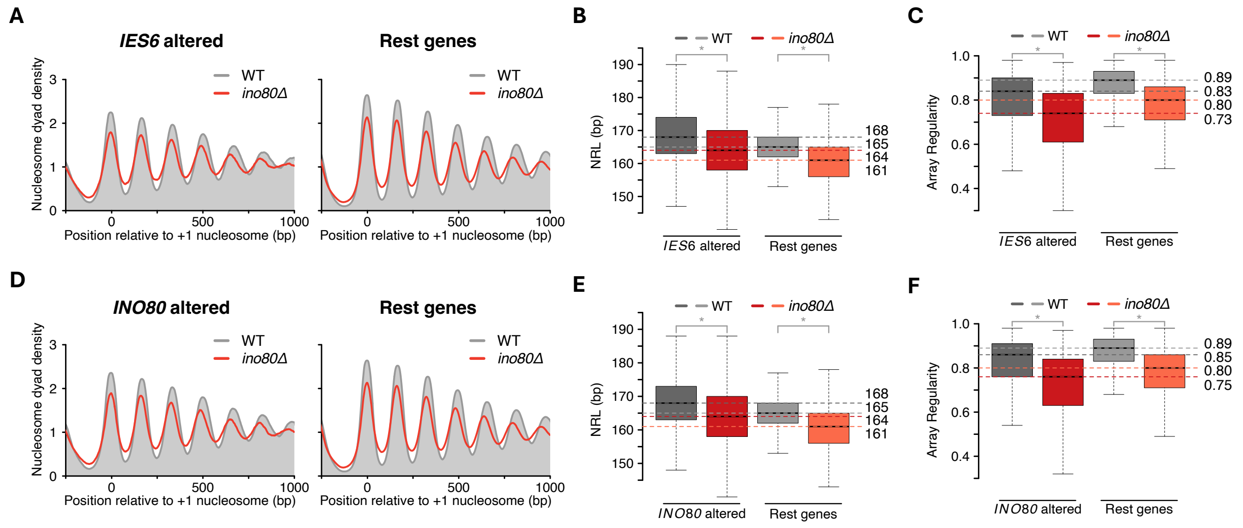

Genes with altered expression do not show different INO80 activity compared to unaltered genes in *ino80Δ* cells. (**A**) Average nucleosome organization in *ino80Δ* cells for all 1304 genes with at least 1.5-fold change in gene expression in *ies6Δ* cells (left) and rest (right) of the genes in the indicated yeast strains. List of differentially expressed genes was obtained from [4]. (**B**) Boxplots showing NRL distribution in the same set of genes as in (A). (**C**) Boxplots showing array regularity distribution in the same set of genes as in (A). (**D**) Average nucleosome organization in *ino80Δ* cells for all 1519 genes with at least 1.5-fold change in gene expression in *ino80Δ* cells (left) and rest (right) of the genes in the indicated yeast strains. List of differentially expressed genes was obtained from [4]. (**E**) Boxplots showing NRL distribution in the same set of genes as in (D). (**F**) Boxplots showing array regularity distribution in the same set of genes as in (D). Horizontal dotted line indicates the median NRL or array regularity in WT (gray shades) and *ino80Δ* (red shades) cells.

Supp. Fig. S6

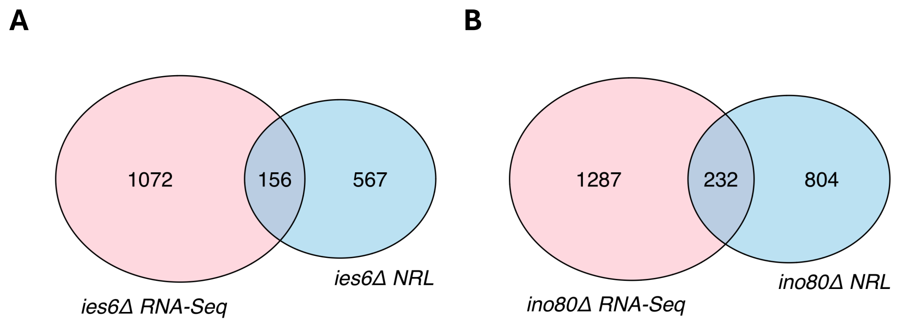

Genes with altered expression and more than 5 bp altered NRL in *IES6* and *INO80* lacking cells show a partial overlap. (**A**) Venn diagram showing overlap of genes with altered RNA levels and >5 bp change in NRL in *ies6Δ* cells. (**B**) Venn diagram showing overlap of genes with altered RNA levels and >5 bp change in NRL in *ino80Δ* cells.

References

1. Singh, A. K., Schauer, T., Pfaller, L., Straub, T. & Mueller-Planitz, F. The biogenesis and function of nucleosome arrays. *Nat Commun* **12**, 7011 (2021).
2. Hsieh, L. J. *et al.* A hexasome is the preferred substrate for the INO80 chromatin remodeling complex, allowing versatility of function. *Molecular Cell* **82**, 2098-2112.e4 (2022).
3. Yao, W. *et al.* The INO80 Complex Requires the Arp5-Ies6 Subcomplex for Chromatin Remodeling and Metabolic Regulation. *Mol Cell Biol* **36**, 979–991 (2016).
4. Xue, Y. *et al.* The Ino80 complex prevents invasion of euchromatin into silent chromatin. *Genes Dev.* **29**, 350–355 (2015).
